# An estrogen response-related signature predicts response to immunotherapy in melanoma

**DOI:** 10.3389/fimmu.2023.1109300

**Published:** 2023-05-12

**Authors:** Min Lin, Tian Du, Xiaofeng Tang, Ying Liao, Lan Cao, Yafang Zhang, Wei Zheng, Jianhua Zhou

**Affiliations:** ^1^ Department of Ultrasound, Sun Yat-Sen University Cancer Center, State Key Laboratory of Oncology in South China, Guangzhou, China; ^2^ Department of Breast Surgery, Sun Yat-Sen University Cancer Center, State Key Laboratory of Oncology in South China, Guangzhou, China

**Keywords:** melanoma, immune checkpoint blockade, gene signature, estrogen, tumor-infiltrating lymphocyte

## Abstract

**Background:**

Estrogen/estrogen receptor signaling influences the tumor microenvironment and affects the efficacy of immunotherapy in some tumors, including melanoma. This study aimed to construct an estrogen response-related gene signature for predicting response to immunotherapy in melanoma.

**Methods:**

RNA sequencing data of 4 immunotherapy-treated melanoma datasets and TCGA melanoma was obtained from open access repository. Differential expression analysis and pathway analysis were performed between immunotherapy responders and non-responders. Using dataset GSE91061 as the training group, a multivariate logistic regression model was built from estrogen response-related differential expression genes to predict the response to immunotherapy. The other 3 datasets of immunotherapy-treated melanoma were used as the validation group. The correlation was also examined between the prediction score from the model and immune cell infiltration estimated by xCell in the immunotherapy-treated and TCGA melanoma cases.

**Results:**

“Hallmark Estrogen Response Late” was significantly downregulated in immunotherapy responders. 11 estrogen response-related genes were significantly differentially expressed between immunotherapy responders and non-responders, and were included in the multivariate logistic regression model. The AUC was 0.888 in the training group and 0.654–0.720 in the validation group. A higher 11-gene signature score was significantly correlated to increased infiltration of CD8+ T cells (rho=0.32, p=0.02). TCGA melanoma with a high signature score showed a significantly higher proportion of immune-enriched/fibrotic and immune-enriched/non-fibrotic microenvironment subtypes (p<0.001)–subtypes with better response to immunotherapy–and significantly better progression-free interval (p=0.021).

**Conclusion:**

In this study, we identified and verified an 11-gene signature that could predict response to immunotherapy in melanoma and was correlated with tumor-infiltrating lymphocytes. Our study suggests targeting estrogen-related pathways may serve as a combination strategy for immunotherapy in melanoma.

## Introduction

Melanoma is an aggressive malignant skin cancer that causes the majority of deaths from skin cancers. Chemotherapies by multiple target therapies have been developed during the last decades to combat molecular defects of melanoma, including BRAF inhibitors vemurafenib and dabrafenib (1). However, although these drugs are highly effective for patients with *^V600^* BRAF-mutated melanomas, which account for approximately half of metastatic melanomas, many patients develop resistance within a relatively short period (2). Melanomas are among the most immunogenic tumors therefore studies of immunotherapy (mostly immune checkpoint blockade [ICB] therapy) for metastatic melanoma have received considerable attention. Immune checkpoint inhibitors such as anti-programmed cell death 1 (anti-PD-1) significantly improve relapse-free survival in patients with resected stage III/IV melanomas (3, 4). However, less than 50% of patients could get tumor regression and long-term durable cancer control under ICB therapy, and chronic immune-related adverse events appear to be more common after ICB therapy (5, 6). Therefore, it is important to identify patients who can benefit from ICB therapy and find new strategies to enhance its effectiveness.

Gender influences the progression of melanoma during all phases, with women showing a lower incidence and lower risk of lymph node invasion and visceral metastases compared to men (7). Clinical data showed that female patients with advanced melanoma may not benefit as much from combination ICB treatment as male patients and estrogen level may serve as an important biomarker associated with ICB therapy response (8). Estrogen/estrogen receptor (ER) signaling influences the tumor microenvironment (TME) and affects the efficacy of ICB treatment in certain tumors (9, 10). The immune cells in the melanoma TME have complex crosstalk with the tumor cells which affects the response to treatments (11). A recent study by Chakraborty et al. highlighted that inhibition of estrogen signaling influences intratumoral macrophage polarization in melanoma, increasing ICB efficacy (12). A former study in lung cancer cells identified the selective estrogen receptor degrader fulvestrant as the top compound that increased tumor sensitivity to immune-mediated lysis (13). All these studies suggest estrogen plays a role in antitumor immunity, and an estrogen response-related signature may predict the response to immunotherapy in melanoma.

The objective of the present study was to construct an estrogen response-related gene signature and evaluate its predictive ability for immunotherapy response in melanoma. Additionally, this study investigated the feasibility of combining endocrine therapy with immunotherapy in melanoma.

## Materials and methods

### Patient data acquisition

RNA sequencing (RNA-seq) data of immunotherapy-treated melanoma were obtained from Riaz et al. (anti-PD-1, N=51, https://github.com/riazn/bms038, Gene Expression Omnibus [GEO] accession number GSE91061) (14), Lauss et al. (adoptive T-cell therapy using tumor-infiltrating lymphocytes, N=25, GEO accession number GSE100797) (15), Hugo et al. (anti-PD-1, N=27, GEO accession number GSE78220) (16), and Van Allen et al. (anti-CTLA4, N=39, www.cbioportal.com, study id skcm_dfci_2015) (17). TCGA melanoma RNA expression data (transcript per million) were downloaded from GEO (accession number GSE62944) (18). Survival data of TCGA were retrieved from Liu et al. (19).

### Differential expression analysis

Differential expression analysis was performed using R package DESeq2 (version 1.30.0) (20) using gene raw counts data from https://github.com/riazn/bms038. Genes with absolute fold change>1.5 and adjusted p-value (FDR) <0.05 were selected as differentially expressed genes. The list of estrogen response-related genes (N=299) was obtained by combining the genes from the Molecular Signatures Database (MSigDB) “Hallmark Estrogen Response Early” and “Hallmark Estrogen Response Late”.

### Pathway analysis

50 Hallmark gene sets were downloaded from the Molecular Signature Database (MsigDB, version 7.5.1). Gene set enrichment analysis (GSEA) was performed using R package clusterProfiler (version 3.18.0) (21). Default settings in GSEA function were used excepted the following parameter: eps=0, seed=12345, and pvalueCutoff=1. Pathways with adjusted p-value <0.05 and normalized enrichment score (NES)>1 or NES<-1 were defined as significantly upregulated or downregulated pathways, respectively.

### Immune cell infiltration analysis

XCell and MCPcounter immune cell enrichment scores were estimated using R package xCell (version 1.1.0) (22) and MCPcounter (version 1.2.0) (23), and gene expression data in transcript per million (TPM) were used as input. Fragments per kilobase per million mapped fragments (FPKM) data of GSE91061 was downloaded from GEO (accession number GSE91061) and transformed to TPM in R. TCGA microenvironment subtypes (immune-enriched/fibrotic [IE/F], immune-enriched/non-fibrotic [IE], fibrotic [F], and desert [D]) were downloaded from Bagaev et al. (n=463) (24). H&E image-based tumor-infiltrating lymphocyte (TIL) percentage estimation was obtained from Saltz et al. (n=383) (25).

### Statistical analysis

All statistical analyses were conducted using R software (version 4.0.3). R package stats (version 4.0.3) was used for univariate and multivariate logistic regression. Gene expression data (log2TPM) of the 4 immunotherapy-treated melanoma datasets and TCGA melanoma cases were first normalized by z-score normalization separately. The immunotherapy response data and the expression levels (z-score) of the 11 estrogen response-related genes from Riaz et al. (14) (GSE91061) were used as the input for the construction of the 11-gene prediction model. The model was then applied to data from Lauss et al. (15), Hugo et al. (16), Van Allen et al. (17) and TCGA (18) to calculate the prediction score. R package “pROC”(version 1.18.0) was used to plot the receiver operating characteristic (ROC) curve and calibration curve (26). Wilcoxon rank-sum test and Chi-squared test were used in the comparison between two groups for continuous and categorical variables, respectively. Log-rank test was used for two-group survival comparison in Kaplan-Meier plot.

## Results

### Estrogen response signatures were downregulated in ICB responders.

**Figure 1:**
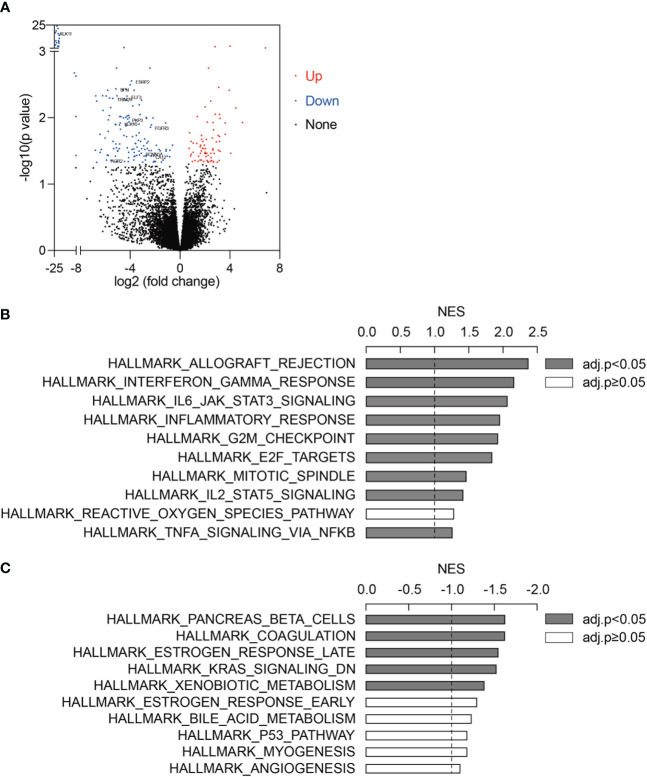
Estrogen response signatures were activated in ICB responders. **(A)** Differentially expressed genes between ICB responders (n=10) and non-responders (n=41). Upregulated genes and downregulated genes (fold change>1.5 and adjusted p-value<0.05) were marked in red and blue, respectively. 11 estrogen response-related genes were labeled. **(B, C)** Top 10 upregulated **(B)** and downregulated **(C)** pathways in ICB responders as compared to non-responders. 50 hallmark gene sets were analyzed using GSEA. Gene sets with adjusted p-value<0.05 were labeled in grey. NES, normalized enrichment score.

Using the pre-treatment RNA-seq data of 51 ICB-treated melanoma (GSE91061), we first performed differential expression analysis between ICB responders (N=10) and non-responders (N=41) (**Supplementary Table 1**). 77 upregulated genes and 155 downregulated genes (fold change>1.5 and adjusted p-value<0.05) were identified in ICB responders as compared to non-responders (**Figure 1A**, **Supplementary Table 2**, **Supplementary Figure 1**). To identify pathways changed between ICB responders and non-responders, we applied gene set enrichment analysis (GSEA) using the 50 hallmark gene sets. Immune-related pathways such as “Hallmark Allograft Rejection”, “Hallmark Interferon Gamma Response”, “Hallmark IL6 JAK STAT3 Signaling”, “Hallmark Inflammatory Response”, and “Hallmark IL2 STAT5 Signaling” were significantly activated in ICB responders (**Figure 1B**, **Supplementary Table 3**). 5 pathways were significantly downregulated in ICB responders (**Figure 1C**, **Supplementary Table 3**), among which “Hallmark Estrogen Response Late” was an estrogen response-related pathway.

### Establishment and evaluation of an 11-gene estrogen response-related signature to predict immunotherapy response

**Figure 2:**
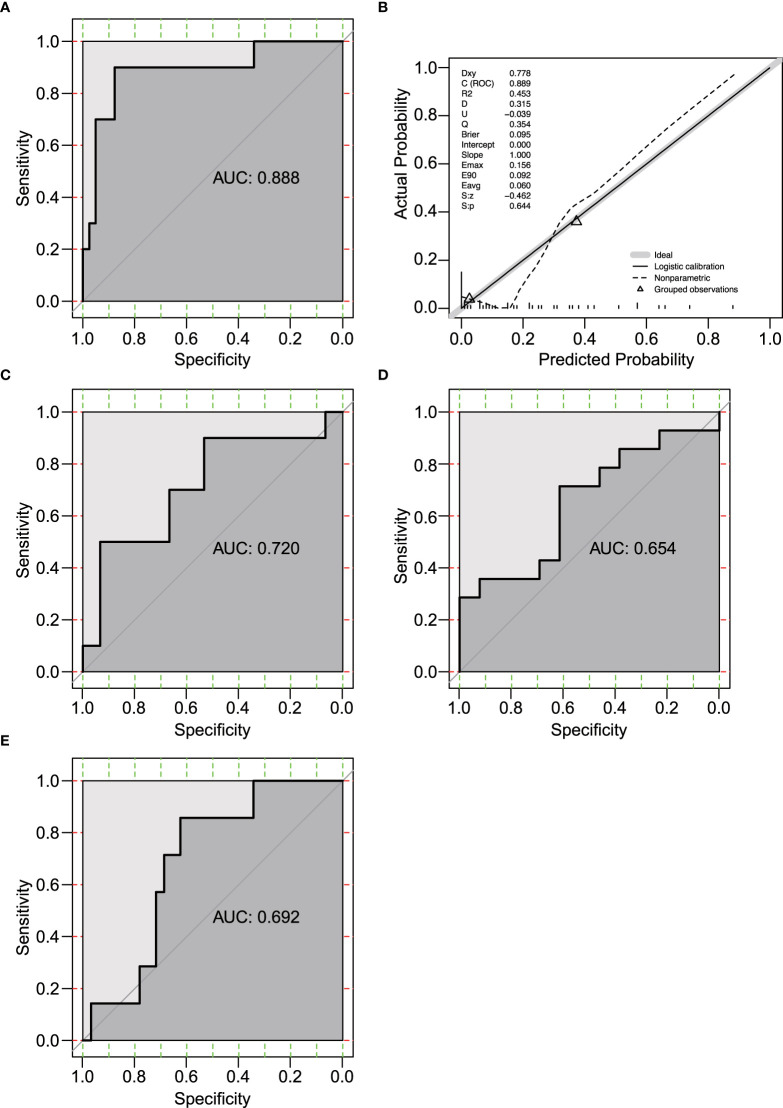
Evaluation of the 11-gene estrogen response-related signature. **(A)** Receiver operating characteristic (ROC) curve of the prediction model in GSE91061 (n=51). **(B)** Calibration curve for the logistic regression model in GSE91061. Dashed line, prediction calibration curve. Solid line, standard curve. **(C-E)** ROC curves of the prediction model in Lauss et al. (n=25) **(C)** (15), Hugo et al. (n=27) **(D)** (16), and Van Allen et al. (n=39) **(E)** (17).

Among the 232 differentially expressed genes between ICB responders and non-responders, 11 genes (AGR2, KLK11, PKP3, ELF3, FGFR3, TRIM29, SFN, KLK10, SCNN1A, CA12, and ESRP2, **Figure 1A**) were estrogen response-related genes. Univariate and multivariate logistic regression showed that none of the 11 genes was significantly associated with ICB response in the 51 patients (**Supplementary Table 4**). Considering estrogen response is a complex pathway involving many genes, we included all 11 genes and developed a multivariate logistic regression model for ICB response in melanoma. The AUC of the prediction model was 0.888 (95% confidence interval 0.786-0.990, by 500 times bootstrap resampling, **Figure 2A**). The calibration curve showed relatively high agreement between the predicted and the actual observation of the ICB responder in the patients with a high predicted probability of being an ICB responder (**Figure 2B**). Besides, heatmap clustering showed the 11-gene signature score could reflect the differentially expressed gene patterns between ICB responders and non-responders (**Supplementary Figure 2**). We further tested the prediction ability of this model using pre-treated RNA expression data of immunotherapy-treated melanoma from Lauss et al. (adoptive T-cell therapy, n=25) (15), Hugo et al. (anti-PD-1, n=27) (16), and Van Allen et al. (anti-CTLA4, n=39) (17) of which the AUCs were 0.720, 0.654 and 0.692 (**Figures 2C–E**), respectively.

### High 11-gene signature score was related to high level of CD8+ T cells and good prognosis

**Figure 3:**
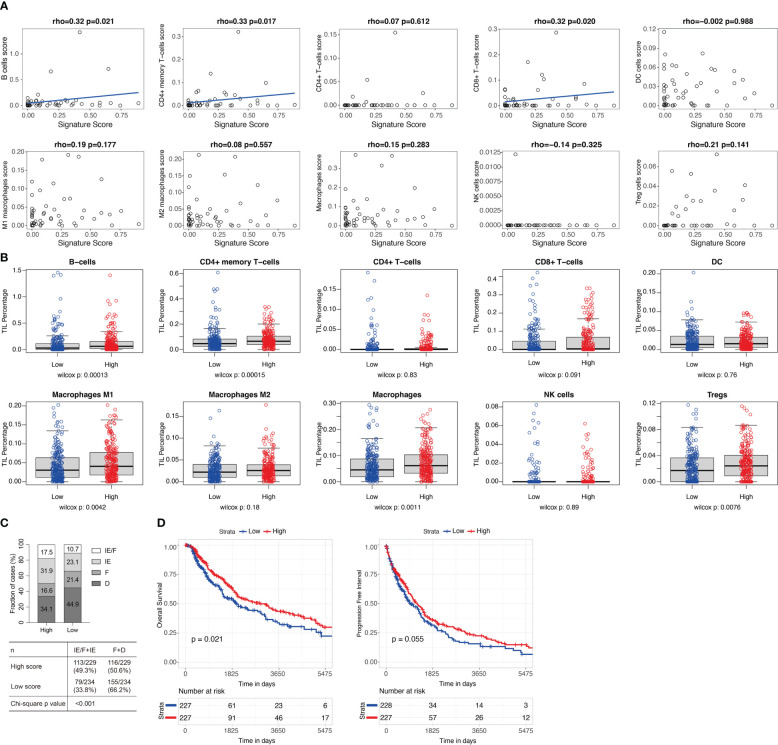
High 11-gene signature score was related to high level of CD8+ T cells and good prognosis. **(A)** Correlations between the 11-gene signature score and the xCell immune cell enrichment scores in GSE91061 (n=51). Xcell immune cell enrichment scores for different types of immune cells were evaluated using R package xCell. Two-sided spearman’s correlation test. A liner regression line was added to help better visualize the correlation using R package ggplot2 for plots with spearman’s correlation test p-values less than 0.05. **(B)** The xCell immune cell enrichment scores for different types of immune cells in TCGA melanoma cases (n=469) with high (n=234) and low (n=235) 11-gene signature scores. Median was used as the cutoff for groups with high and low signature scores. Two-sided Wilcoxon rank-sum test. **(C)** Microenvironment subtypes of TCGA melanoma cases with high (n=229) and low (n=234) 11-gene signature scores (median as cutoff). Four microenvironment subtypes (immune-enriched/fibrotic [IE/F], immune-enriched/non-fibrotic [IE], fibrotic [F], and desert [D]) were downloaded from Bagaev et al. (24). 463 of the 469 melanoma cases in **(B)** had microenvironment subtypes. Percentages of each subtype in the high and low signature score groups were labeled. Chi-squared test. **(D)** Overall survival and progression-free interval of TCGA melanoma cases with high and low 11-gene signature scores (median as cutoff). Log-rank test.

Estrogen response was reported to play an important role in the tumor microenvironment (TME) (27). To study the pathways related to the 11-gene signature score, we performed DE analysis using the median 11-gene signature score as the cutoff for the 51 patients in GSE91061. GSEA showed that “Hallmark Interferon Gamma Response”, “Hallmark Allograft Rejection”, “Hallmark Myogenesis”, “Hallmark Interferon Alpha Response”, and “Hallmark Inflammatory Response” were significantly upregulated in the high-signature score group (**Supplementary Table 5**). As expected, “Hallmark Estrogen Response Late” was downregulated in the high-signature score group (**Supplementary Table 5**). We further tested the correlations of the 11-gene signature score to xCell and MCPcounter immune cell enrichment scores. We found that the 11-gene signature score was significantly correlated with xCell scores of B cells (rho=0.32, p=0.021), CD4 memory T cells (rho=0.33, p=0.017), and CD8+ T cells (rho=0.32, p=0.02) (**Figure 3A**, **Supplementary Table 6**), while no significant correlation was identified between the 11-gene signature score and MCPcounter scores (**Supplementary Figure 3A**). As GSE91061 dataset has a limited number of patients, we then evaluated the relations of the 11-gene signature score to immune cells in TCGA melanoma cases (n=469). Patients with high 11-gene signature scores had significantly increased xCell scores of B cells (p<0.001), CD4+ memory T-cells (p<0.001), M1 macrophages (p=0.004), macrophages (p=0.001), and Tregs (p=0.0076), and a trend of high CD8+ T-cells score (p=0.091) (**Figure 3B**, **Supplementary Table 7**). Using MCPcounter scores, we also found higher scores of B lineage (p<0.05), monocytic lineage (p<0.05), CD8+ T cells (p=0.07), and cytotoxic lymphocytes (p=0.06) in TCGA high 11-gene signature score group (**Supplementary Figure 3B**, **Supplementary Table 7**). A Higher TIL percentage (p=0.078) was also identified in TCGA high 11-gene signature score group using H&E image-based TIL data (**Supplementary Figure 3C**) (25). Bagaev et al. (24) identified four microenvironment subtypes (immune-enriched/fibrotic, immune-enriched/non-fibrotic, fibrotic, and desert) in TCGA patients, among which the immune-enriched/fibrotic and immune-enriched/non-fibrotic subtypes had a higher level of immune activation and better response to ICB as compared to the other two subtypes. We also observed a significantly higher proportion of immune-enriched/fibrotic and immune-enriched/non-fibrotic subtypes (49.3% *vs* 33.8%, chi-squared test, p-value<0.001) in the TCGA melanoma cases with high 11-gene signature score (**Figure 3C**). Besides, TCGA melanoma cases with a high 11-gene signature score had significantly better progression-free interval (p=0.021) and a tendency towards better overall survival (p=0.055) (**Figure 3D**).

### Inhibition of estrogen signaling may contribute to better ICB response in melanoma

**Figure 4:**
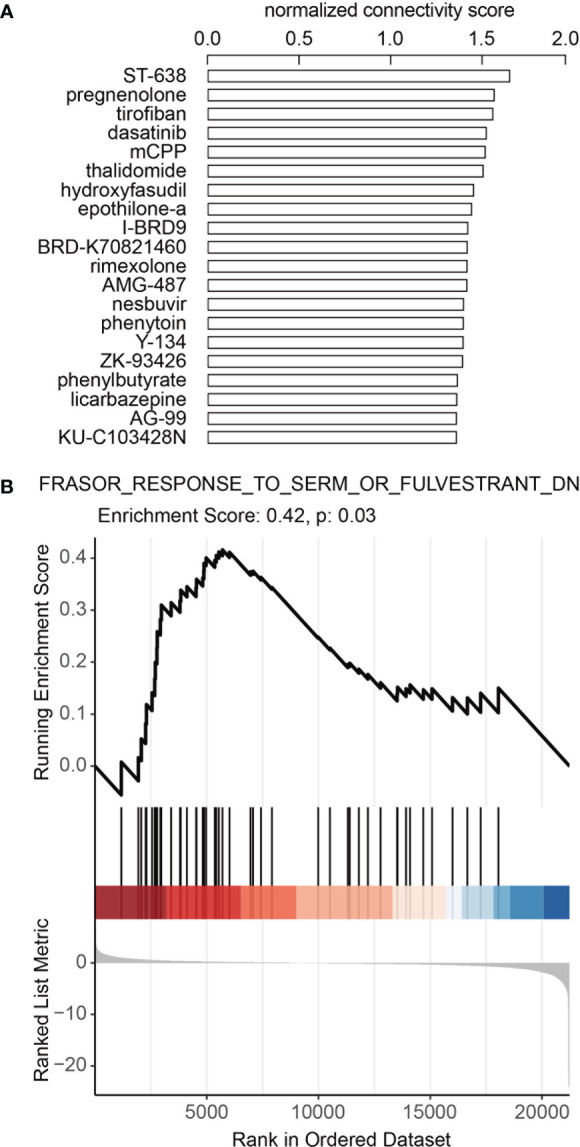
Inhibition of estrogen signaling may contribute to better ICB response in melanoma. **(A)** Top 20 candidate drugs which may turn melanoma from ICB non-responder to responder. Connectivity map (CMap) Touchstone datasets was queried to identify drugs that could induce similar gene expression pattern as the DE gene expression profile between ICB responders and ICB non-responders in GSE91061. Adjusted p-values<0.05 for all drugs. **(B)** Downregulated gene signature by SERM or SERD was enriched in melanoma ICI-responders. GSEA analysis was performed in GSE91061 using signature “Frasor Response to SERM or Fulvestrant DN (downregulated genes)”.

To identify potential drugs that could turn melanoma from ICB non-responders to ICB responders, we queried Connectivity map (CMap) Touchstone datasets for drugs that induced similar gene expression patterns as the DE gene expression profile between ICB responders and ICB non-responders in GSE91061. In melanoma cell line-A375, we found that an estrogen receptor antagonist, Y-134 was among the top 20 drugs which had the most similar (ranked by normalized connectivity score, FDR<0.05) induced gene expression pattern as the DE genes described above (**Figure 4A**, **Supplementary Table 8**), and an estrogen agonist, DY-131 had negative connectivity score (FDR<0.05), which indicates the induced gene expression pattern of this drug was opposing to our input DE gene expression profile (**Supplementary Table 8**). Further GSEA using previously published gene signature by selective estrogen receptor modulator (SERM) or selective estrogen receptor degrader (SERD) (28) showed that downregulated gene signature by SERM or SERD was enriched in melanoma ICB-responders (**Figure 4B**, **Supplementary Figure 4**, **Supplementary Table 9**). All these data suggesting combining ICB with the inhibition of estrogen signaling may lead to improved ICB response in melanoma.

## Discussion

Melanoma accounts for the majority of deaths from skin cancer. For non-resectable/metastatic melanoma, much progress has been made in targeted therapy and immunotherapy. However, there is still a lack of practical prognostic markers. Estrogen receptors are broadly expressed in many cell types involved in the innate and adaptive immune responses and play a potential immune regulatory role in the TME (29). A recent study elucidated estrogen signaling influences intratumoral macrophage polarization in melanoma and consequently enhances ICB therapy efficacy (12). These findings suggest that, estrogen response-related genes can potentially be predictive markers for ICB therapy response in melanoma. In the current study, using estrogen response-related genes that were differentially expressed between ICB responders and non-responders in melanoma, we constructed an 11-gene immunotherapy response prediction signature with stable predictive performance in different melanoma datasets treated with multiple types of immunotherapies (anti-PD-1, anti-CTLA-4 and adoptive T-cell therapy). This signature was also significantly correlated with the infiltration of multiple types of tumor immune cells and was prognostic for overall survival in melanoma.

Estrogen and estrogen response-related genes play a modulatory role in melanoma progression, probably through influencing antitumor immunity. Melanoma incidence has a gender divergence. Slightly higher rates of melanoma have been reported for young women (20-45 years) which subsequently decrease after 45 years of age (30). On the contrary, melanoma incidence progressively increases in males after 50 years of age (31). An increased risk of melanoma was associated with early age at menarche and late age at menopause, which highlighted the role of sex steroid hormones in melanoma (32). Estrogen signaling accelerates the progression of different estrogen-insensitive tumor models by contributing to deregulated myelopoiesis by both driving the mobilization of myeloid-derived suppressor cells (MDSC) and enhancing their intrinsic immunosuppressive activity (33). A study by Chakraborty et al. revealed that estrogen signaling affects intratumoral macrophage polarization in melanoma and increased ICB efficacy (12). ERβ has been reported to be the predominant ER subtype in melanoma and could represent a marker for metastatic potential and prognosis (34). ERβ activation might impair melanoma development through the inhibition of the PI3K/Akt pathway and displays a protective role in the metastatic process (34, 35). In this study, GSEA revealed that estrogen response-related pathway “Hallmark Estrogen Response Late” was significantly downregulated in ICB responders. These findings indicate the potential of estrogen response-related gene signature in predicting ICB therapy response in melanoma.

The prognostic model proposed in the present study was composed of 11 estrogen response-related genes (AGR2, CA12, ELF3, ESRP2, FGFR3, KLK10, KLK11, PKP3, SFN, SCNN1A, TRIM29), among which many genes correlated with tumor immunity and immunotherapy. Carbonic anhydrase 12 (CA12) mediated the survival of macrophages in relatively acidic TME, while on the other hand, it induced macrophage production of large amounts of C-C motif chemokine ligand 8 (CCL8), which enhanced cancer cell epithelial-mesenchymal transition and facilitated tumor metastasis (36). CA12 was included in a former gene signature for predicting the prognosis of uveal melanoma (37). Fibroblast growth factor receptor 3 (FGFR3) is among the receptor tyrosine kinases which may be activated *via* autocrine circuits in melanoma (38). FGFR3 is a biomarker of immune infiltration and immunotherapy response of bladder cancer (39). Kallikrein-related peptidases (KLKs) have been reported to possess novel functions in innate immunity and inflammation. KLK10 is expressed in the follicular dendritic cells that are essential for the maturation of B cells (40). KLK10 was dynamically regulated in T cells *in vitro* in response to viral antigens and in activated monocytes, pointing to its activities in the development of adaptive and innate immune function (41). Plakophilin 3 (PKP3) encodes a component of desmosomes with mechanical barrier function in the skin and other normal tissues. PKP3 expression was revealed to be markedly elevated in melanoma metastasis lacking immune gene signature and was strongly associated with decreased patient survival (42). SCNN1A encodes the α subunit of epithelial sodium channel and was reported to be relevant to tumor progression in a variety of cancers (43). Lou et al. revealed that SCNN1A involves in tumor immune process by influencing tumor immune cell infiltration (44). TRIM29 is a negative regulator of NK cell functions (45). TRIM29 expression was higher in patients with higher TIL and proved to be related to immune dysfunction in colorectal cancer (46). In this study, the 11-gene signature score showed a correlation with tumor immune infiltration, which may be the basis of its predictiveness in the response to different immunotherapies.

In this study, we found that estrogen receptor antagonist Y-134 could induce a similar gene expression pattern as the DE gene expression profile between ICB responders and non-responders while estrogen agonist DY-131 could induce an opposite gene expression pattern. This discovery suggests the possibility of using anti-estrogen therapy to enhance the efficacy of immunotherapy. Accumulating evidence from experimental and clinical studies has revealed the multifaceted immunomodulatory effects of endocrine therapies, especially in the modulation of TME. SERM like tamoxifen and raloxifene could affect the functional differentiation and immunostimulatory capacity of dendritic cells (47). CARMINA 02 trial assessed 86 pre- and post-neoadjuvant endocrine therapy (NET) tumor samples and found significantly increased TIL numbers in post-NET samples of responders (48). Preclinical studies indicated that SERD, a class of ERα antagonists, interacts with ER-positive immune cells in the TME such as MDSCs, TILs, and other selected immune cell subpopulations. SERD-induced inhibition of MDSCs and concurrent actions on CD8+ and CD4+ T-cells promote the interaction of immune checkpoint inhibitors with breast cancer cells and augment the curative effect (10). In melanoma, inhibition of estrogen signaling affects intratumoral macrophage polarization and increased ICB efficacy (12). Our GSEA using previously published gene signatures by SERM or SERD also showed that downregulated gene signature by SERM or SERD was enriched in melanoma ICB-responders. These findings raise the possibility of using anti-estrogens as an approach to enhance the effectiveness of ICB therapies in melanoma, but further research is needed.

There are several limitations to this study. Firstly, the datasets in this study were all retrospective data of small sample sizes from public databases. More prospective real-world data are needed to verify the clinical utility of this model. Secondly, the conclusions obtained were based on bioinformatics analysis and require further validation *in vivo* and *in vitro*. Thirdly, the use of solely estrogen response-related genes to build a prognostic model may have excluded other prognostic genes in melanoma, which limits the overall predictive capacity of the model. Finally, this model did not incorporate other risk factors and clinical parameters that may impact prognosis, which may lead to underestimating the true prognostic capacity of the model.

In conclusion, in this study, we identified and verified an 11-gene signature that could predict response to immunotherapy in melanoma. This model was a prognostic factor for melanoma patients and was correlated with TIL. The results of this study provide new perspectives regarding the potential strategies for combining immunotherapy with endocrine therapy for treating melanoma patients.

## Data availability statement

Publicly available datasets were analyzed in this study. This data can be found here: https://www.ncbi.nlm.nih.gov/geo/query/acc.cgi?acc=GSE91061 https://www.ncbi.nlm.nih.gov/geo/query/acc.cgi?acc=GSE100797 https://www.ncbi.nlm.nih.gov/geo/query/acc.cgi?acc=GSE78220 https://www.ncbi.nlm.nih.gov/geo/query/acc.cgi?acc=GSE62944 http://www.cbioportal.org/study/summary?id=skcm_dfci_2015.

## Author contributions

TD and ML designed the experiment. TD and ML analyzed and interpreted the data. X-FT, LC, YL and Y-FZ assisted with the data and figure integration. TD, X-FT provided methodology, reviewing, and editing. WZ and J-HZ conceived and supervised the study. ML wrote the manuscript with all authors' contributions to writing and providing feedback. WZ and J-HZ acted as guarantors and corresponding authors for this study. All authors contributed to the article and approved the submitted version.

## Supplementary material

Supplementary Figure 1 Schematic showing the construction and evaluation of the 11-gene estrogen response related ICB response prediction signature.

Supplementary Figure 2 Heatmap of the differentially expressed genes showing clustering of the 11-gene signature score high and low groups. Z-scores of differentially expressed genes from were used. Heatmap was plotted using R function heatmap.3. Canberra distance between samples was calculated and hclust=ward was used in the hierarchical clustering. ICB responders and non-responders were labeled with black and grey. Patients with high and low 11-gene signature scores (median as cutoff) were labeled with red and blue. The 11 genes used in the final signature were labeled in yellow.

Supplementary Figure 3
**(A)** Correlations between the 11-gene signature score and the MCPcounter immune cell enrichment scores in GSE91061 (n=51). MCPcounter immune cell enrichment scores for different types of immune cells were evaluated using R package MCPcounter. Two-sided spearman’s correlation test. A liner regression line with a confidence interval of 0.95 was added to help better visualize the correlation using R package ggplot2. Of note, the slope of the calculated line by ggplot2 “geom_smooth” function (method=“lm”) may not be perfectly matched to the spearman’s rank correlation coefficient (rho). **(B)** The MCPcounter immune cell enrichment scores for different types of immune cells in TCGA melanoma cases (n=469) with high (n=234) and low (n=235) 11-gene signature scores. Median was used as the cutoff for groups with high and low signature scores. Two-sided Wilcoxon rank-sum test. **(C)** H&E image-based tumor-infiltrating lymphocyte (TIL) percentage of TCGA melanoma cases with high and low 11-gene signature scores (median as cutoff, high, n=199; low, n=184).

Supplementary Figure 4 GSEA plot of “Frasor Response to SERM or Fulvestrant Up (upregulated genes)”.

Supplementary Table 1 Clinical characteristics of melanoma cases used in the study.

Supplementary Table 2 List of differentially expressed genes between ICB responders and non-responder in GSE91061.

Supplementary Table 3 GSEA between ICB responders and non-responders in GSE91061 using 50 hallmark gene sets.

Supplementary Table 4 Univariate and multivariate analyses of the 11-estrogen response-related genes in GSE91061.

Supplementary Table 5 GSEA between high and low 11-gene signature score groups in GSE91061 using 50 hallmark gene sets.

Supplementary Table 6 Correlations between the 11-gene signature score and the xCell immune cell enrichment scores in GSE91061.

Supplementary Table 7 Correlations between the 11-gene signature score and the xCell and MCPcounter immune cell enrichment scores in TCGA melanoma cases.

Supplementary Table 8 List of drugs that could induce significant (adjusted p.value<0.05) similar (or inverse) gene expression pattern as the DE gene expression profile between ICB responders and ICB non-responders in GSE91061.

Supplementary Table 9 List of genes in “Frasor Response to SERM or Fulvestrant Up” and “Frasor Response to SERM or Fulvestrant Down”.
