# Supplementary material for: An estrogen response-related signature predicts response to immunotherapy in melanoma
Source: Front Immunol. 2023 May 12;14:1109300. doi: 10.3389/fimmu.2023.1109300 (PMC10213284; doi:10.3389/fimmu.2023.1109300)
Supplement: Supplementary Figure 1 — Schematic showing the construction and evaluation of the 11-gene estrogen response related ICB response prediction signature. [file DataSheet_1.zip › Supplementary materials/Supplementary table 4.docx]

**Supplementary table 4 Univariate and multivariate logistic regression of 11 estrogen response-related genes**

| **Characteristics** | **Univariate** | | **Multivariate** | |
| --- | --- | --- | --- | --- |
|  | **OR** | ***P*** | **OR** | ***P*** |
| AGR2 | -1.075 | 0.453 | -4.667 | 0.0563 |
| KLK11 | -0.939 | 0.356 | -1.393 | 0.5794 |
| PKP3 | -1.093 | 0.228 | 1.540 | 0.6198 |
| ELF3 | -0.299 | 0.503 | 0.900 | 0.653 |
| FGFR3 | -1.023 | 0.096 | -0.631 | 0.7919 |
| TRIM29 | -2.757 | 0.318 | -2.182 | 0.0809 |
| SFN | -3.870 | 0.179 | 0.910 | 0.8358 |
| KLK10 | -2.192 | 0.349 | -7.402 | 0.0884 |
| SCNN1A | -0.457 | 0.384 | -2.811 | 0.3683 |
| CA12 | -0.847 | 0.073 | 4.199 | 0.212 |
| ESRP2 | -0.317 | 0.480 | -1.068 | 0.2144 |
