# Supplementary figures and images for: An estrogen response-related signature predicts response to immunotherapy in melanoma
Source: Front Immunol. 2023 May 12;14:1109300. doi: 10.3389/fimmu.2023.1109300 (PMC10213284; doi:10.3389/fimmu.2023.1109300)

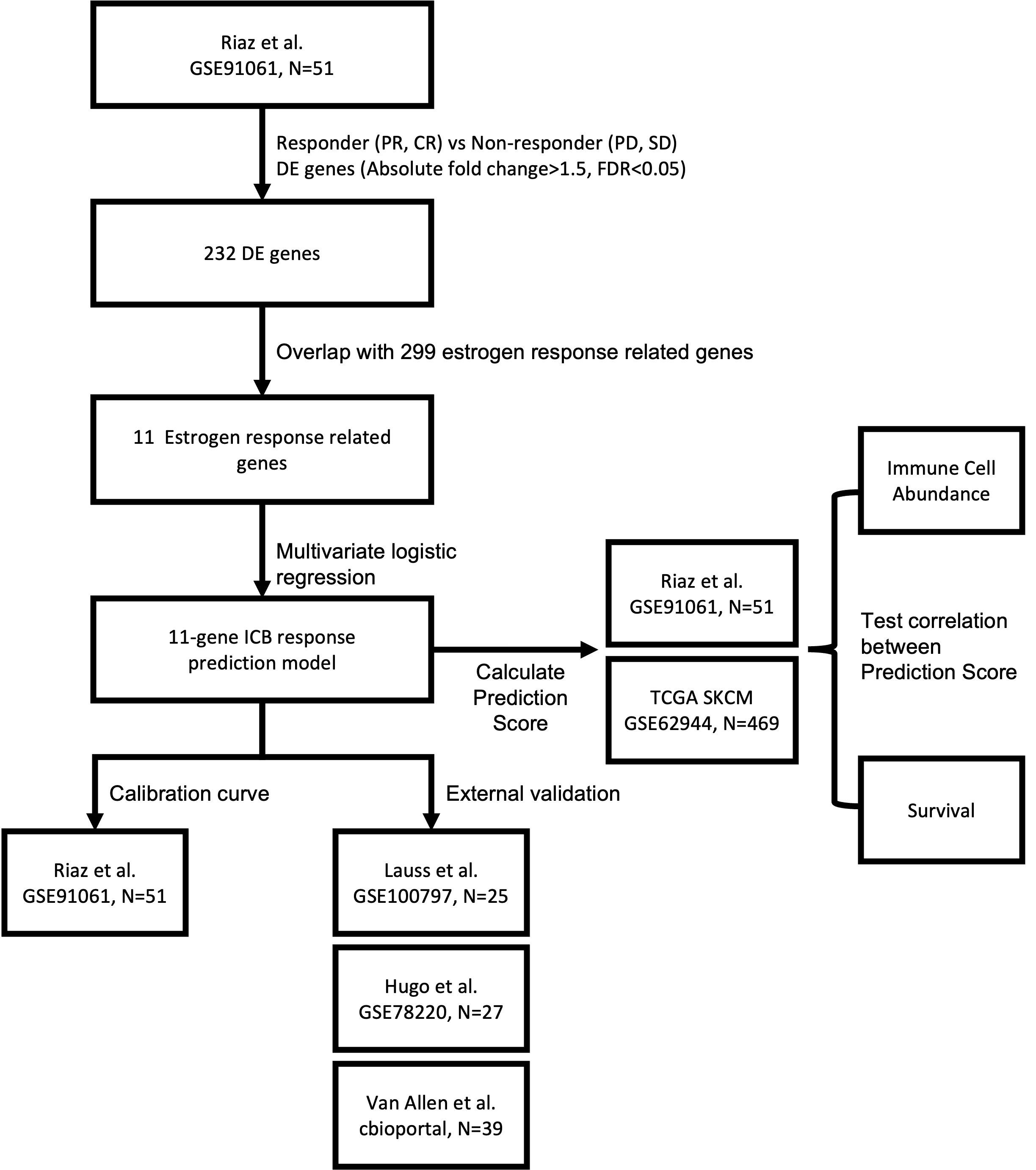

Supplement: Supplementary Figure 1 — Schematic showing the construction and evaluation of the 11-gene estrogen response related ICB response prediction signature. [file DataSheet_1.zip › Supplementary materials/Supplementary Figure 1.jpg]
